# Results of the Cologne Corona Surveillance (CoCoS) study – a cross-sectional study: survey data on risk factors of SARS-CoV-2 infection in adults

**DOI:** 10.1186/s12889-023-15047-6

**Published:** 2023-02-06

**Authors:** Max Oberste, Nadja Schnörch, Kija Shah-Hosseini, Teodora Asenova, Felix Dewald, Clara Lehmann, Michael Buess, Gerd Fätkenheuer, Florian Klein, Kerstin Daniela Rosenberger, Annelene Kossow, Florian Neuhann, Martin Hellmich

**Affiliations:** 1 Institute of Medical Statistics and Computational Biology, Medical Faculty and University Hospital of Cologne, University of Cologne, Robert-Koch-Straße 10, 50931 Cologne, Germany; 2 Institute of Virology, Medical Faculty and University Hospital of Cologne, University of Cologne, Fürst-Pückler-Straße 56, 50935 Cologne, Germany; 3 Department of Internal Medicine, Medical Faculty and University Hospital of Cologne, University of Cologne, Kerpener Str. 62, 50931 Cologne, Germany; 4 Cologne Health Authority, Cologne, Germany; 5 Institute of Hygiene, University Hospital of Muenster, University Muenster, Robert-Koch-Straße 49, 48149 Muenster, Germany; 6 Heidelberg Institute of Global Health, University, Heidelberg, Germany; 7 School of Medicine and Clinical Sciences, Levy Mwanawasa Medical University, Lusaka, Zambia

**Keywords:** Risk association, Self-reported infection, Compliance to countermeasures, Age, Social factors

## Abstract

**Background:**

The personal, environmental, and behavioral risk factors that play an important role in the spread of SARS-CoV-2 are still largely unclear. At the same time, there is limited evidence on the effectiveness of specific countermeasures for SARS-CoV-2. As a first approach to these questions, we use data from the Cologne Corona Surveillance (CoCoS) study, a large cross-sectional study conducted in Cologne, Germany, in June 2021.

**Methods:**

This study was conducted in Cologne, Germany. Six thousand randomly selected Cologne residents who were 18 years of age or older were invited to participate in this study. Participant information was obtained via an online survey. Previous SARS-CoV-2 infections were recorded using self-reports. Sociodemographic and environmental information such as age, sex, living situation were collected. Potential SARS-CoV-2 risk behaviors were captured (workplace situation, adherence to hygiene regulations, and regular use of public transportation). Adherence to hygiene regulations was surveyed by determining the compliance with the ‘AHA’-rules (German acronym that stands for keeping a distance of 1.5 m from fellow citizens, hand disinfection, and wearing a face mask). Binary logistic regression analysis was used to identify risk factors for SARS-CoV-2 infection.

**Results:**

A sample of 2,433 study participants provided information. Comparison of the sample with the general population showed representativeness for most sociodemographic characteristics with a preference for higher level of education in the study sample. Younger age, as well as living with minor children (under 18 years) in the same household were associated with a higher number of self-reported SARS-CoV-2 infections. Adherence to hygiene regulations was associated with fewer self-reported SARS-CoV-2 infections in adults. Gender, size of living space per person, workplace situation (work from home versus working with contact to colleagues/customers), and regular use of public transportation showed no significant association with self-reported SARS-CoV-2 infections in multivariable analysis.

**Conclusion:**

The presented results provide initial indications of which sociodemographic and behavioral factors may be associated with SARS-CoV-2 infection. However, the fact that these factors were recorded without exact dates and could have changed accordingly during the pandemic or after infection limits the strength of the results.

**Trial registration:**

DRKS.de, *German Clinical Trials Register* (DRKS), Identifier: DRKS00024046, Registered on 25 February 2021.

**Supplementary Information:**

The online version contains supplementary material available at 10.1186/s12889-023-15047-6.

## Introduction

The SARS-CoV-2 pandemic has resulted in a global health crisis. More than 570 million people worldwide tested positive for SARS-CoV-2 at the time of writing in August 2022 [1]. Meanwhile, German health authorities have reported over 30 million cases and over 144,000 SARS-CoV-2-related deaths [2].

In order to prevent an overload of the health care system, the federal government of Germany initiated countermeasures against the spread of SARS-CoV-2. Citizens had to severely limit their social contacts [3]. This also included the occupational sector. Employees were encouraged to work from home or to minimize contact with customers and colleagues [4]. A closure of public schools and kindergartens was implemented over long periods [5]. At times, further closures, for example restaurants and retail were initiated [3] and curfews imposed. It was strongly recommended to follow the applicable hygiene regulations, which, in addition to social distancing, included washing hands regularly and wearing a face mask [5]. Local public transport was maintained to a reduced extent [6].

Groups with a higher risk for severe courses of illness were mainly defined by older age and pre-existing health conditions [7]. Those individuals were entitled to protective measures like an early supply of face masks, transportation services to avoid public traffic, and prioritized access to early vaccinations [6].

Implementing countermeasures came with the price of serious psychological, social and economic burden [8]. At the same time, the effectiveness of specific countermeasures and their interrelation remains largely unclear and little evidence is publicly available to this day. As a first approach to these questions, we use data from the Cologne Corona Surveillance (CoCoS) study, a large cross-sectional study conducted in Cologne, Germany, in March and June 2021 [9]. The aim was to identify associations between specific sociodemographic and behavioral factors and the cumulative prevalence of SARS-CoV-2 infection.

## Methods

**Fig. 1:**
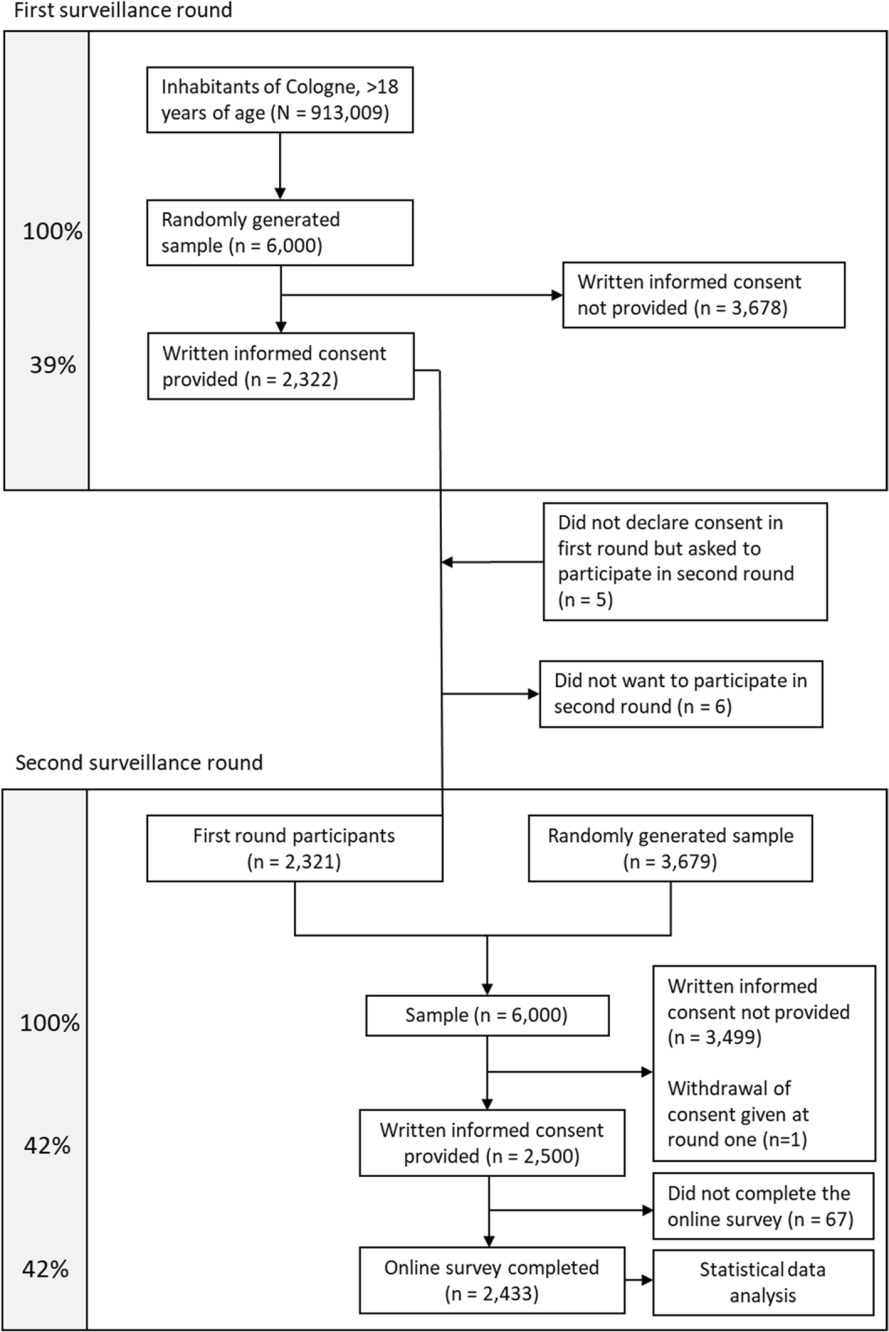
Flow chart of enrollment and written consent in each surveillance round

A detailed description of the CoCoS study design can be found elsewhere [1]. First results of the CoCoS study including saliva probes have been published [10]. Participants had to be citizens of Cologne 18 years of age or older and were identified using municipal registration. In the official registration management program (MESO, HSH Soft- und Hardware Vertriebs GmbH, Ahrensfelde OT Lindenberg, Germany) a random sample of citizens meeting the inclusion criteria for inclusion in the database was drawn using a generator. The original study consisted of two surveillance rounds, the first in March 2021 and the second in June 2021. We present the data from the second surveillance round. The study design thus corresponds to a cross-sectional study design. A flowchart of the enrollment of study participants is shown in Fig. 1.

## Setting

Cologne is located in the federal state of North Rhine-Westphalia and is the fourth largest city in Germany. The city has 1.1 million inhabitants of 183 different nationalities. Administratively, Cologne is organized into nine districts and 86 sub-districts. Of note, 17.5% of the population belong to the vulnerable age group (over 65 years) for SARS-CoV-2-infections [11]. Meanwhile, over 413,000 SARS-CoV-2-cases have been officially reported in Cologne [12]. Locally reported cases of SARS-CoV-2 infections were primarily congruent with the numbers throughout Germany [12]. The citizens of Cologne were subjected to SARS-CoV-2-countermeasures according to instructions of the federal state authorities of North Rhine-Westphalia and their local implementation by a municipal crisis unit [5].

## Questionnaire

The participants received the invitation to our study by mail. Besides a request for written consent, the invitation letter contained a QR code with authorization to participate in the online survey. The questionnaire was designed similarly to the ELISA and MuSPAD questionnaires developed by the ‘Helmholtz Zentrum für Infektionsforschung, Braunschweig, Germany’ [13]. Firstly, the online survey requested basic information such as age and gender. Participants were asked if they received a positive SARS-CoV-2 test result in the past. This question did not include COVID-19 self-tests but asked explicitly for PCR-test confirmed infection. Subjects could answer ‘yes’, ‘no’ or ‘do not specify’ as response options here. If subjects selected ‘Yes’, the follow-up question presented the following options: ‘I had a positive test result at least once’, ‘I had negative test results only’, ‘test result not known' or ‘no indication’. This strategy of questioning was intended to indicate all PCR test confirmed infections known to the subjects themselves. The questionnaire also included questions about participants’ living conditions. Participants were asked about the size of the living space per person and the number of minor children in the household. For data analysis the variable ‘age’ was stratified into the categories 18 to 44, 45 to 64, and 65 years of age or older with the latter as reference category. Living space per person was divided into categories ‘less than 25m^2^ per person’, ‘25 to 50m^2^ per person’, and ‘more than 50m^2^ per person’ (reference category). The variable ‘number of minor children in household’ was subdivided into the categories ‘no children under 18 years in household’, ‘one child under 18 years in household’, and ‘two or more children under 18 years in household’ because only 45 participants stated that they had three children or more.

In the following section, data considering potential risk behaviors of the participants was collected. These included the workplace situation, adherence to hygiene regulations, and regular use of public transportation. With regard to workplace situation, participants were asked about the use of ‘working from home’ and contact with employees or customers at work. The variable ‘workplace situation’ included the categories ‘working from home or at working without contact to customers or colleagues’, ‘at work with contact to customers and/or colleagues’, and ‘not working’ (reference category). Adherence to hygiene regulations was defined as adherence to the so-called ‘AHA-rules’. The acronym 'AHA' stands for the German initials of the words for keeping your distance, washing/disinfecting your hands and wearing a face mask. The acronym 'AHA' was also used in official language in Germany to communicate hygiene regulations [4] and should have been widely known accordingly. Participants could indicate whether or not they followed the AHA-rules. The regular use of public transportation was queried by asking subjects to indicate whether they regularly used public transportation in the period from February 1, 2020 (the start of the pandemic) to the day of the survey. Participants were given the choice of answering: 'Yes, I regularly used public transport during this period' or 'No, I did not regularly use public transport during this period'. Regular use was counted if the participant reported to frequently use one of the vehicles 'bus', 'train', or 'cab' in the given time period.

It was assumed that uncertainties about online questionnaires occur disproportionately often in the group of older participants, and to prevent distortions here, the option of conducting the questionnaire in the form of a telephone interview was set up.

## Statistical analysis

Absolute and relative frequencies of key sociodemographic characteristics of the sample of 6,000 potential participants were determined using data from the municipal registration office. Absolute and relative frequencies of the more in-depth sociodemographic information from the questionnaire were determined from the participant sample. The sociodemographic information of potential participants and the participant sample were compared with the official statistics of the city of Cologne on its adult general population [11].

Binary logistic regression analysis was used to identify associations between the surveyed sociodemographic and behavioral factors and self-reported SARS-CoV-2 infection. The following sociodemographic and behavioral factors were considered: age, gender, living space per person, number of minor children in household, workplace situation, adherence to hygiene regulations (AHA-regulations) and use of public transportation. Univariable logistic regression was fitted for each variable. Before multivariable logistic regression was carried out, multicollinearity was assessed by examining variance inflation factors (VIFs) of all included variables. Any variable with VIF larger than five was supposed to be excluded.

All variables were initially included in the multivariable analysis. In the case of the variables 'Age' and 'Living space per person', which were evaluated univariably both continuously and discretely, the continuous variable was included in the multivariable analysis, since it provides more information than the variables divided into categories. Stepwise backward selection based on *p* > 0.05 (Wald statistic) was used for exclusion to identify relevant associations.

All reported *p*-values are 2-sided and were considered statistically significant if below 0.05. We did not adjust for multiple testing due to the explorative and descriptive character of this study. Calculations and figures were carried out using SPSS Statistics (IBM Corp., Armonk, NY, USA) and Excel (Microsoft Corp., Redmond, WA, USA), as well as R (R Foundation for Statistical Computing, Vienna, Austria).

## Results

### Study recruitment and population

The flow chart of study recruitment is depicted in Fig. 1. In the first round of CoCoS study in March 2021, 6,000 participants were invited. Participants who had completed the first round were invited again for the second round in June 2021. The number of participants who had completed the first surveillance round and were therefore invited to the second round was 2,321 corresponding to 38.7%. Therefore, 3,679 new participants were additionally invited. From this resulting sample of 6,000 for the second round, 2,500 provided written consent and 2,433 who completed the online survey will be used to examine SARS-CoV-2 risk factors using their information given at the time of June 2021.

### Description of study participants

A detailed description of the study population’s sociodemographic characteristics is provided in Table 1. The study population was reasonably representative of the general adult population regarding gender, age, and size of household. However, the participants in the CoCoS study showed a higher level of education than the general population of Cologne regarding high school graduation (55.00%/75.14%). Moreover, the participants with citizenship other than German were underrepresented with 5.87% in the study whereas 20.32% of the general adult population of Cologne have a citizenship other than German. Not least, the study participants reported fewer SARS-CoV-2 infections with 3.45% (95% CI 2.73%-4.18%) compared to 5.06% (95% CI 3.60%-5.61%) officially recorded by Cologne health authorities in June 2021. In addition, the vaccination rate of the study sample was significantly higher with 79.95% receiving at least one vaccination in comparison to 66.23% of the general adult population receiving at least one vaccination by June 18th, 2021. At the same time, the rate of unvaccinated individuals in the study sample was lower compared to the general adult population (33.76%/20.05%).

**Table 1:** Sociodemographic characteristics and SARS-CoV-2 specific information of the CoCoS study collective (survey completed, *n* = 2,433) compared to the potential participants and the general adult Cologne population

	General adult Cologne population		Potential participants		CoCoS study sample (survey completed)	
**Sample**						
Participants (18 yrs. or older)	913,009	100%	6,000	100%	2,433	40.55%
**Gender**^a^						
Female	470,577	51.54%	3,086	51.44%	1,312	53.93%
**Age**^a^						
18–34 years (%)	270,538	29,63%	1,662	27.70%	570	23.43%
35–59 years old (%)	388,678	42,57%	2,640	44.01%	1,125	46.24%
60–74 years old (%)	153,890	16,86%	1,095	18.25%	511	21.00%
75 years or older (%)	99,903	10,94%	602	10.04%	227	9.33%
**No. of household members**						
Average	913,009	1.88	NA	NA	1,906	2.32
1–2	438,859	77.68%	NA	NA	1,302	68.31%
3–4	106,314	18.82%	NA	NA	535	28.07%
5 or more	19,800	3.50%	NA	NA	69	3.62%
Missing values^b^	-	-	-	-	527	21.66%
**School education**						
No school leaving certificate	36,520	4.00%	NA	NA	9	0.46%
Secondary school diploma	374,333	41.00%	NA	NA	475	24.39%
High school graduation	502,155	55.00%	NA	NA	1,463	75.14%
Missing values^b^	-	-	-	-	486	19.97%
**Employment status**						
Student/apprenticeship	122,849	13.46%	NA	NA	178	8.90%
Employed	582,613	63.81%	NA	NA	1,153	57.71%
Self-employed	NA	NA	NA	NA	226	11.31%
Retired	NA	NA	NA	NA	326	16.32%
Unemployed	45,225	4.60%	NA	NA	49	2.45%
Other^c^	NA	NA	NA	NA	66	3.30%
Missing Values^b^	-	-	-	-	435	17.88%
**Primary citizenship**^a^						
German	727,503	79.68%	5,039	84.00%	2,290	94.12%
Other than German	185,506	20.32%	960	16.00%	143	5.87%
**Cumulative SARS-CoV-2 cases**						
Start of the pandemic until June 18, 2021	46,195	5.06%	NA	NA	84	3.45% [2.73%-4.18%]
**Vaccination rate until June 18, 2021**						
Vaccinated at least once	604,692	66.23%	NA	NA	1,591	79.95%
Vaccinated twice	351,674	38.52%	NA	NA	672	33.77%
Not vaccinated	308,308	33.76%	NA	NA	399	20.05%
Missing Values^b^	NA	NA	NA	NA	443	18.20%

^a^Information obtained directly from the population register

^b^While the percentages on the variables expressed relate to the respondents of the respective questionnaire item, the percentages on the missing values relate to the sample

^c^Maternity leave, parental leave, parental leave or other leave of absence, retraining, federal voluntary service, voluntary social/ecological year

### Analysis of associations between the surveyed sociodemographic and behavioral factors and SARS-CoV-2 infection

**Fig. 2:**
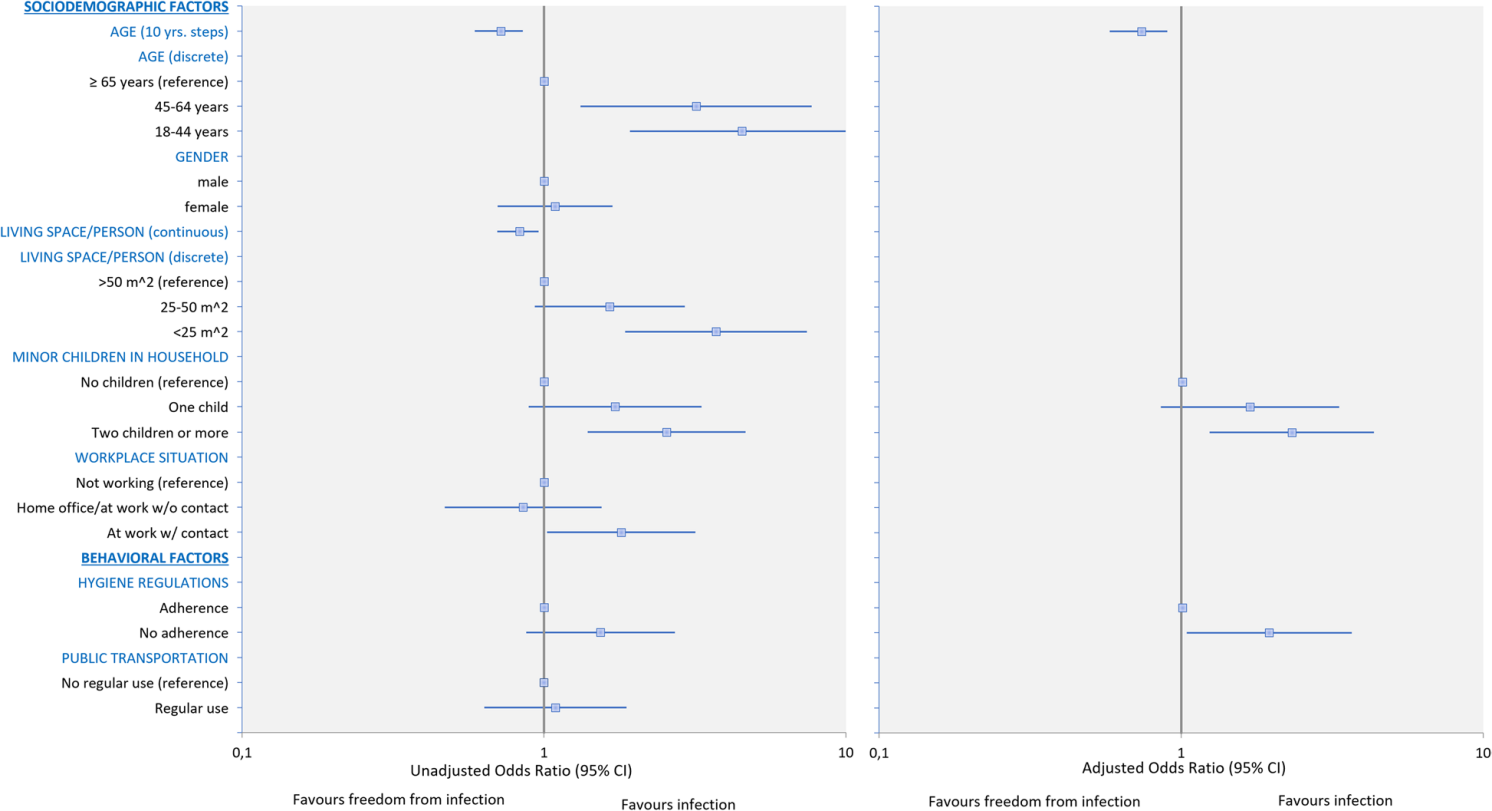
Forest plots of the results of univariable and multivariable logistic regression. **A** Univariable risk factor analysis, **B** Multivariable risk factor analysis, CI: confidence interval

**Fig. 3:**
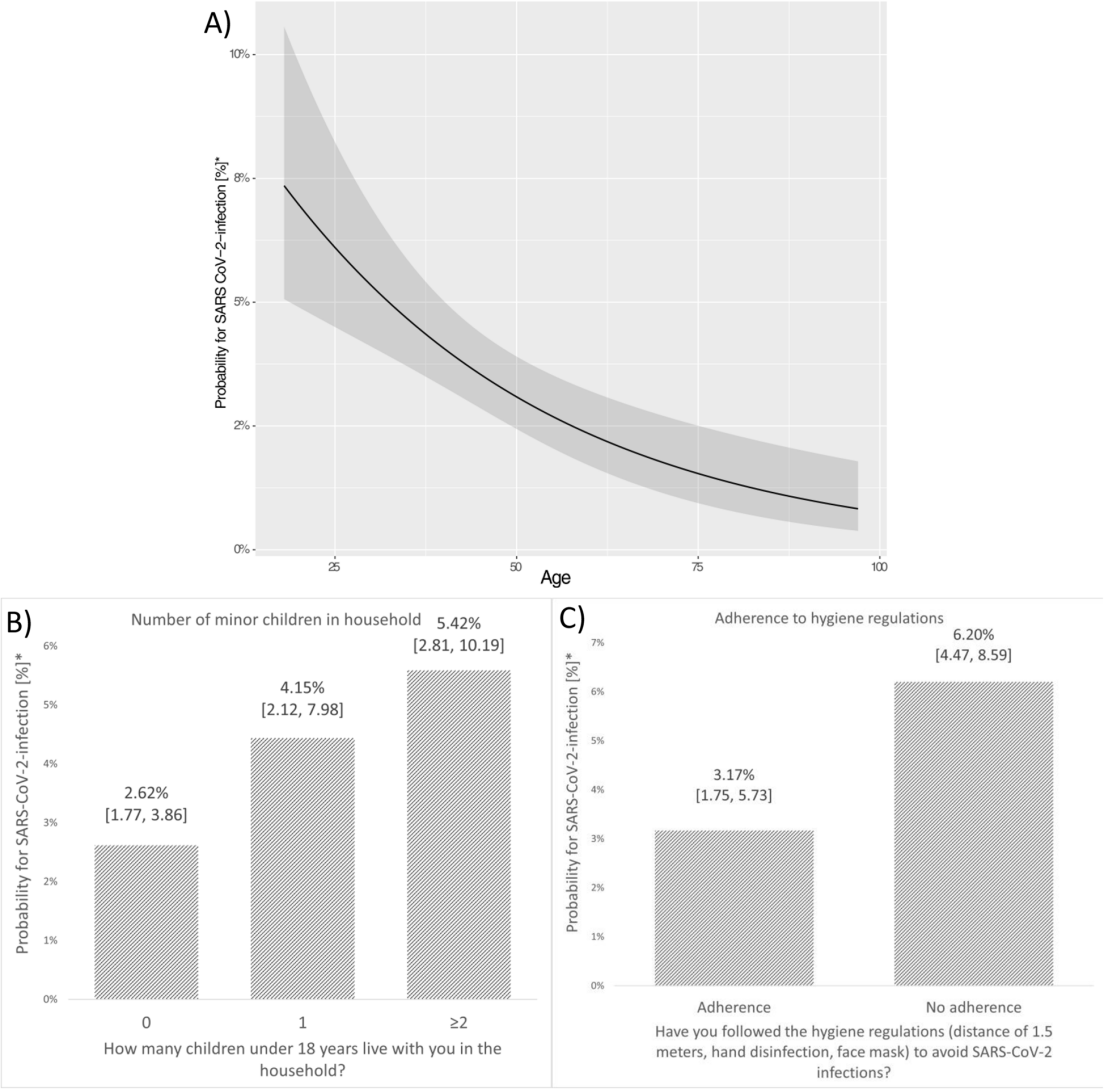
Estimated marginal means of probabilities for SARS-CoV-2 infection from multivariable logistic regression with stepwise backward selection (Wald statistic as criterion). *Calculated as estimated marginal means from multivariable analysis. **A** Probability for SARS-CoV-2 infection [%] depending on predictor variable ‘age’ (continuous). **B** Probability for SARS-CoV-2 infection [%] depending on predictor variable ‘number of minor children in household’. **C** Probability for SARS-CoV-2 infection [%] depending on predictor variable ‘adherence to hygiene regulations’

Data of 2,433 participants were included in the statistical analysis. Eighty-four participants reported a previous infection with SARS-CoV-2 (3.45%; CI 2.73–4.18). The results of both univariable and multivariable logistic regression analyses are presented in Table 2 and depicted in Fig. 2. Estimated marginal means of probabilities for SARS-CoV-2 infection from multivariable logistic regression are shown in Fig. 3. In the multivariable analysis, 1,794 participants could be included. The multicollinearity test did not reveal any indication to take further steps, all predictors had a VIF smaller than 1.4. The results of the multicollinearity assessment are included in the Supplementary Material to this article. Sensitivity analysis using the subset of participants for multivariable analysis on univariable analyses is also provided in the Supplementary Material to this article.

**Table 2:** Results of univariable and multivariable logistic regression

Variables		Univariable analysis (*N* = 2,433)			Multivariable analysis (*N* = 1,794)		
	No. of events/total no. (%)	Odds	Odds Ratio	p	Adjusted Odds*	Adjusted Odds Ratio	p
Total	84/2433 (3.5)	0.036 [0.029, 0.044]					
Sociodemographics							
Age *(continuous)*			0.972 [0.959, 0.985]	< 0.001		0.974 [0.959, 0.990]	0.002
Age *(discrete)*				0.002			
≥ 65 years	6/546 (1.1)	0.011 [0.005, 0.035]					
45–64 years	31/906 (3.4)	0.035 [0.025, 0.051]	3.189 [1.322, 7.693]	0.010			
18–44 years	47/981 (4.8)	0.050 [0.038, 0.067]	4.529 [1.924, 10.663]	0.001			
Gender				0.705			
Male	37/1121 (3.3)	0.034 [0.025, 0.047]					
Female	47/1312 (3.6)	0.037 [0.028, 0.050]	1.089 [0.702, 1.687]	0.705			
Livings space per person *(continuous)*			0.983 [0.970, 0.996]	0.010			
Livings space per person *(discrete)*				0.001			
> 50 m^2^	17/652 (2.6)	0.027 [0.017, 0.043]					
25–50 m^2^	42/993 (4.2)	0.044 [0.032, 0.060]	1.650 [0.931, 2.924]	0.086			
< 25m^2^	17/188 (9.0)	0.099 [0.060, 0.164]	3.713 [1.857, 7.427]	< 0.001			
Number of minor children in household				0.005			0.016
No minor children in household	59/2014 (2.9)	0.030 [0.023, 0.039]			0.029 [0.018, 0.040]		
One minor child in household	11/223 (4.9)	0.052 [0.028, 0.095]	1.719 [0.889, 3.324]	0.107	0.044 [0.022, 0.087}	1.651 [0.839, 3.249]	0.147
Two children or more minor children in household	14/196 (7.1)	0.077 [0.045, 0.132]	2.549 [1.396, 4.654]	0.002	0.057 [0.029, 0.113]	2.368 [1.272, 4.408]	0.007
Behavioral factors							
Workplace situation				0.013			
Not working	20/564 (3.5)	0.037 [0.024, 0.057]					
Work from home/at work w/o contact	25/823 (3.0)	0.031 [0.021, 0.047]	0.852 [0.469, 1.550]	0.600			
At work with contact	34/547 (6.2)	0.066 [0.047, 0.094]	1.803 [1.024, 3.173]	0.041			
Adherence to hygiene regulations							
Adherence	15/604 (2.5)	0.025 [0.015, 0.043]			0.037 [0.018, 0.057]		
No adherence	69/1829 (3.8)	0.039 [0.031, 0.050]	1.539 [0.874, 2.712]	0.135	0.062 [0.045, 0.086]	2.145 [1.163, 3.958]	0.015
Public transportation							
No regular use	67/1973 (3.4)	0.025 [0.015, 0.043]					
Regular use	17/460 (3.7)	0.039 [0.031, 0.050]	1.092 [0.635, 1.877]	0.751			

^*^Calculated as estimated marginal means with weighted mean for categorical factors and mean age (47.39 years)

^*^The first variable in each category is used as reference unless otherwise specified

#### Associations between sociodemographic factors and SARS-CoV-2 infection

The univariable analysis revealed that younger age was significantly associated with a higher number of SARS-CoV-2 infections (OR 0.972, 95% CI: 0.959–0.985, *p* < 0.001). Accordingly, the percentage of participants reporting an infection was the lowest within the age group of over65 years with 1.1% (6/546) compared to 3.4% (31/906) within the age group 45–64 years and 4.8% (47/981) within the age group 18–44 years. Compared to reference age group of over 65 years, participants in the age group 45–64 years (OR 3.189, 95% CI: 1.322–7.693, *p* = 0.010) and in the age group 18–44 years (OR 4.529, 95% CI: 1.924–10.663, *p* = 0.001) were significantly less likely to report past SARS-CoV-2 infections. The association between younger age and more SARS-CoV-2 infections also persisted in the multivariable model. Multivariable logistic regression showed a significant decrease in the odds of infection by 2.6% per year of age (OR 0.974, 95% CI: 0.959–0.990, *p* = 0.002). Gender did not show to be significantly associated with SARS-CoV-2 infection, neither in univariable nor in multivariable logistic regression (*p* = 0.705).

In univariable analysis, a significantly higher probability of self-reported infection with SARS-CoV-2 was found for smaller living space per person (OR 0.983, 95% CI: 0.970–0.996, *p* = 0.010). A percentage of 2.6% (17/652) of participants reported a SARS-CoV-2 infection in the category living in > 50m^2^ per person, 4.2% (42/993) in the category living in 25-50m^2^ per person and 9.0% (17/188) in the category living in less than25m^2^ per person. Participants in the category with less than 25m^2^ were significantly more likely to report past SARS-CoV-2 infections than the reference category of participants with more than 50 m^2^ of living space (OR 3.713, 95% CI: 1.857–7.427, *p* < 0.001). Interestingly, the association between square meters per person of living space and SRAS-CoV-2 infection was no longer statistically significant in the multivariable logistic regression. The variable was excluded from the model during stepwise backward selection.

The number of minor children in household was found to be significantly associated with the number of reported SARS-CoV-2 infections in univariable analysis (*p* = 0.005). Among participants living without minor children in the household, 2.9% (59/2014) reported that they had undergone a SARS-CoV-2 infection. Among those living with one minor child in the household, 4.9% (11/223) reported past SARS-CoV-2 infections, and those living with two or more minor children in the same household, 7.1% (14/196) reported past SARS-CoV-2 infections. Participants living with two children or more were statistically significant more likely to report past SARS-CoV-2 infections than the reference category of participants without minor children in household in univariable analysis (OR 2.549, 95% CI: 1.396–4.654, *p* = 0.002). This pattern also persisted in the multivariable model (*p* = 0.016, comparison between ‘two or more children’ and ‘no children’: OR 2.326, 95% CI: 1.243–4.354, *p* = 0.008).

#### Associations between behavioral factors and SARS-CoV-2 infection

Univariable analysis identified a significant association between workplace situation and reported SARS-CoV-2 infections (*p* = 0.013). The workplace situation with work from home or at work without contact to customers or colleagues showed a percentage of 3.0% (25/823) reported infections compared to 6.2% (34/547) reported infections in the workplace situation with regular contact to customers and colleagues. At the same time, the not-working reference group reported 3.5% (20/564) of infections. The greater odds of participants with regular contact to customers and colleagues to report past SARS-CoV-2 infections compared to reference group of not-working individuals reached statistical significance in univariable analysis (OR 1.803, 95% 1.024–3.173, *p* = 0.041). However, in the multivariable analysis, the significant association between the situation at work and reported past SARS-CoV-2 infections did not hold. The variable was excluded from the model during stepwise backward selection.

Adherence to hygiene regulations (adherence to the widely implemented AHA-rules) was associated with a lower probability of SARS-CoV-2 infection. In the adherent group, a percentage of 2.5% (15/604) of participants reported an infection compared to 3.8% (69/1829) in the non-adherent group. This result failed to reach statistical significance in univariable analysis using the entire sample (*n* = 2,433, *p* = 0.135). However, sensitivity analysis using the subset of participants for multivariable analysis on univariable analysis (see supplementary material to this article) showed significant influence of adherence to hygiene regulations (*p* = 0.018). In the multivariable analysis, non-adherence to hygiene regulation was found to be significantly associated with a higher number of SARS-CoV-2 infections (OR 1.957, 95% CI 1.043, 3.669, *p* = 0.036) compared to adherence to hygiene regulations. Whether or not participants regularly used public transportation was not significantly associated with reported past SARS-CoV-2 infections, neither in univariable (*p* = 0.751) nor in multivariable logistic regression. A joint description based on the actual data of the high-risk group with younger age, minor children living in the same household, and no adherence to hygiene recommendations is provided in the supplementary material to this article.

## Discussion

This study provides data on cumulative self-reported SARS-CoV-2 infections in the adult Cologne population from the beginning of the pandemic until June 2021. Combined with a questionnaire on sociodemographic characteristics and behaviors, the study provides first indications of which sociodemographic characteristics and which behaviors might be associated with a higher number of SARS-CoV-2 infections. Our results show that younger people are more likely to acquire SARS-CoV-2 infection than older people. In addition, the results show an association between self-reported SARS-CoV-2 infection and living with minor children (under 18 years) in the household, as well as non-adherence to common hygiene regulations (AHA-regulations). At the same time, multivariable analysis showed no statistically significant association between self-reported SARS-CoV-2 infection and gender, living space per person, workplace situation, and regular use of public transportation (regular use of bus, train, or cab).

The model derived from the survey results shows that cumulative cases decrease sharply with age. Similar studies have confirmed that older age is associated with a lower prevalence of SARS-CoV-2 [14]. A possible explanation for this finding is that older people are more risk averse, having a higher likelihood of severe course of illness and higher mortality [15]. Studies show that older people indeed reported to be more careful and adherent to protective measures [16]. Furthermore, the decrease of infections by year of age could also be influenced by early vaccinations in the older population [14]. Although much less common, younger adults can also become seriously ill with COVID-19. In addition, numerous infections in the younger population can lead to a faster spread of SARS-CoV-2 [17]. One way to reduce the number of infections among younger people could be to increase awareness campaigns explicitly targeting this age group. This is already being addressed in the campaign presented by the German Federal Ministry of Health in October 2022, in which younger testimonials also emphasize the importance of protecting against the pandemic. The data of the present study do not provide any indications as to which aspects such campaigns should particularly address in order to reach younger people especially well. Future studies should explore these questions in order to make campaigns as effective as possible.

The finding that living with minor children in the same household is significantly associated with more self-reported SARS-CoV-2 infections of adults should be further investigated in future studies. It should be noted that similar studies have also reached other conclusions that argue against an increased risk for adults living with minor children in the household [17]. A comparison between the risk of living with other adults and the risk of living with minors would allow a more nuanced risk evaluation, since living with minor children entails different daily chores and external exposures. If it is confirmed that living with children favors infections, measures such as additional sick leave for parents, as the German government has done for 2021, would certainly be a useful relief for families.

Adherence versus non-adherence to hygiene recommendations did not prove to be significant in the univariable model, but it did prove to be significantly associated with SARS-CoV-2 infection in the multivariable model. Wearing face masks, hand disinfection and keeping a 1.5 m distance from fellow citizens have been enforced by the German government (commonly known in Germany by the acronym ‘AHA-rules’, which stands for the first letters of the German words for keep your distance, wash your hands and wear a face mask) since early in the pandemic in 2020 [6]. In line with similar previous findings [17], adherence to the hygiene measures known as ‘AHA-rules’ in Germany were associated with fewer self-reported SARS-CoV-2 infections in the examined study population. However, it should not be ignored that people who follow the AHA rules may also be more likely to adopt broader preventive measures, such as general avoidance of social contact. Future studies should capture these further prevention strategies and analyze the effectiveness of adherence to the AHA rules in the context of these broader prevention strategies.

In the presented study population, gender was not associated with the cumulative prevalence of self-reported SARS-CoV-2 infection among the participants (*p* = 0.705). This was different in other studies that found a slightly increased risk of infection among male participants [17]. Those differences could be explained by varying sociodemographic characteristics in different study samples. Therefore, the effect of gender should be further investigated in upcoming studies.

Moreover, previous studies have shown that lesser living space per person increases the risk of SARS-CoV-2 infection [18]. In a recent study of our group, a lower socio-economic status was shown to be associated with an increased risk for SARS-CoV-2 infection [18]. However, in the current study, statistically significant association between less living space per person and more SARS-CoV-2 infections was only detected in the univariable logistic analysis indicating interactions with other living conditions such as children in the household.

Compared to the unemployed reference group, participants in the regular workplace situation with customer and coworker contact reported a much higher percentage of SARS-CoV-2 infections. This result was significant in univariable logistic regression. According to the ‘SARS-CoV-2 Occupational Health and Safety’ ordinance from January 21, 2021 [4], general measures such as wearing a face mask, regular ventilation in closed rooms, and frequent SARS-CoV-2 testing were required in the regular workplace situation [5]. Therefore, protection against SARS-CoV-2 should have been provided to a certain degree in the regular work situation with customer and employee contact, although it remains unclear to what degree these specified measures were actually implemented. Work from home and work without interpersonal contact showed a lower percentage and lower association with SARS-CoV-2 infection. In addition, the effect of working from home in reducing infection risk has been observed in similar studies [17]. Results regarding the risk of infection at the regular workplace were published, especially about occupations with much SARS-CoV-2 contact showing a higher risk of infection [19]. It must be mentioned that not every occupational group has the opportunity of working from home, such as health care workers. Additionally, the association was no longer apparent in the multivariable logistic regression. This could be due to interactions with other included variables.

The here presented analyses come with strengths and limitations. A strength of the study is its large, representative sample. The participation rate of this study is comparable to most cohort studies with multiple conduction rounds in Germany [20]. The questionnaire covered a wide range of areas and created a detailed picture of the participant’s sociodemographic profile and behaviors potentially associated with SARS-CoV-2 infection. A detailed analysis of the estimated SARS-CoV-2 incidence in this study sample has been published elsewhere [10]. However, the cumulative SARS-CoV-2 cases in the study population are 3.45% (95% CI 2.73–4.18). This is less than the official figures in this period, which were 5.06% (95% CI 3.60%-5.61). The difference could be explained by sociodemographic differences in the study sample with a higher acceptance of measures to contain COVID-19 compared to the general population resulting in fewer infections. Future conduction periods could aim at a higher participation rate, especially in underrepresented sociodemographic groups more likely to report a SARS-CoV-2 infection [18].

The sociodemographic characteristics of the sample indicate differences to the general adult population of Cologne in some respects. The sample shows a clear preference for a higher level of education, which can be associated with a higher socio-economic status. This assumption can be supported by a significantly lower percentage of unemployment in the study sample (4.6% in the general Cologne population versus 2.45% in the sample). These selection effects might have favored a lower estimated incidence in the sample. A recent study of our group shows that districts of Cologne with a lower socio-economic status reported more cumulative SARS-CoV-2 cases from February 2020 to October 2021[18].

Since the presented cumulative SARS-CoV-2 cases are based on questionnaire data, the unreported number of asymptomatic infections discovered by blood sampling cannot be determined. Another relevant limitation of our study is the survey of infections in the population aged 18 and older. Results based on household information showed a significant association between children in the household and SARS-CoV-2 infection. This association could be further investigated by surveying the estimated seroprevalence of children and adolescents under 18 years of age. Furthermore, our study did not include institutionalized individuals who were shown to have a more SARS-CoV-2 infections at the given period [21].

The study design entails the risk of selection effects, which is particularly evident in the study group regarding school leaving certificate and higher education. Since we did not offer monetary compensation, the participation in several implementation rounds could be motivated by an increased sense of social responsibility, also leading to a higher adherence to countermeasures and regulations. Especially adherence to the hygiene regulations was significantly associated with fewer self-reported SARS-CoV-2 infections, both in univariable and multivariable logistic regression. Therefore, selection of risk-aware individuals and those with higher education could have led to observing a decreased number of SARS-CoV-2 infections in the study population.

The study primarily observed associations between the presence or absence of self-reported SARS-CoV-2 infection in relation to sociodemographic and behavioral factors. Severity of the course of infection should be included in the examination of infection risk in future studies.

The categories of variables, such as the situation at work, are very broad due to the exploratory nature of the study. There is a risk that participants with very different characteristics and behaviors are subsumed into the same category despite the inherent differences between professions in terms of interpersonal contact. This fact may have biased the results of the present study. Given the fairly high level of education of our sample, it is more likely that office work or similar occupations predominated among the participants. Future studies should go into further detail here in order to assess more accurately the associations with SARS-CoV-2 infection posed by the situation in the workplace.

The fact that the participants could only answer yes or no regarding adherence to hygiene regulations represents a simplification of the realities. Gradations, such as a 5-point Likert scale with the options 'never', 'rarely', 'sometimes', 'often' or 'always' can significantly increase the information content in future studies. The same applies to the joint consideration of the various hygiene measures. Individual observations of the three different measures would have provided more detailed insights.

The definition of regular use of public transport is imprecise. The term regular is not clearly defined and could have been interpreted differently between participants. Future studies can increase the information value by asking about the use of public transport with the answer options of, for example, a 5-point Likert scale from 'never' to 'daily'.

Associations between behavioral factors and cumulative prevalence must be interpreted with caution, as it is unclear whether behavior may have changed as a result of a positive test result or infection. Future surveys should ask more clearly about behavior before a possible infection. Last but not least, it should also be noted that we did not correct for multiple testing due to the exploratory and descriptive nature of our analysis.

## Conclusion

Despite selection bias the study provides important insights into regional associations between SARS-Cov-2 infection and sociodemographic and behavioral factors. In the presented study, adherence to hygiene regulations (AHA) was significantly associated with fewer self-reported SARS-CoV-2 infections. Whether this result is an indication of the effectiveness of corresponding measures remains open due to the cross-sectional nature of the study. In addition, younger age and living with minor children in the household was significantly associated with self-reported SARS-CoV-2 infection. The presented results can help identify constellations with higher risk of acquiring SARS-CoV-2 infection and therefore target and communicate protective measures.

## Supplementary Information

**Additional file 1.** 

## Data Availability

The datasets used and/or analysed within the current study are available from the corresponding author upon reasonable request.

## Abbreviations

DRKS: German Clinical Trials Register
SARS-CoV-2: Severe Acute Respiratory Syndrome-Coronavirus-2
CoCoS-study: Cologne Corona Surveillance Study
COVID-19: Corona Virus Disease 19
