# Supplementary material for: Results of the Cologne Corona Surveillance (CoCoS) study – a cross-sectional study: survey data on risk factors of SARS-CoV-2 infection in adults
Source: BMC Public Health. 2023 Feb 6;23:260. doi: 10.1186/s12889-023-15047-6 (PMC9902063; doi:10.1186/s12889-023-15047-6)
Supplement: Supplementary file 1 — Additional file 1. [file 12889_2023_15047_MOESM1_ESM.docx]

**S1 Sensitivity analysis showing univariable logistic regression with entire sample and only with complete cases used in multivariable logistic regression**

|  | **Univariable analysis (N = 2433)** | | | | **Univariable analysis (N = 1794)** | | | |
| --- | --- | --- | --- | --- | --- | --- | --- | --- |
| **Variables** | **No. of events/total no. (%)** | **Odds** | **Odds Ratio** | **p** |  | **Odds** | **Odds Ratio** | **p** |
| **Total** | 84/2433 (3.5) | 0.036 [0.029, 0.044] |  |  | 74/1794 | 0.043 [0.035, 0.053] |  |  |
| **Sociodemographics** |  |  |  |  |  |  |  |  |
| **Age *(continuous)*** |  |  | 0.972 [0.959, 0.985] | <.001 |  |  | 0.975 [0.960, 0.990] | .001 |
| **Gender** |  |  |  | .705 |  |  |  | .685 |
| Male | 37/1121 (3.3) | 0.034 [0.025, 0.047] |  |  | 32/817 (3.9) | 0.041 [0.029, 0.058] |  |  |
| Female | 47/1312 (3.6) | 0.037 [0.028, 0.050] | 1.089 [0.702, 1.687] | .705 | 42/977 (4.3) | 0.045 [0.033, 0.061] | 1.102 [0.689, 1.762] | .685 |
| **Livings space per person *(continuous****)* |  |  | 0.983 [0.970, 0.996] | .010 |  |  | 0.984 [0.971, 0.997] | .014 |
| **Number of minor children in household** |  |  |  | .005 |  |  |  | .030 |
| No children | 59/2014 (2.9) | 0.030 [0.023, 0.039] |  |  | 49/1398 (3.5) | 0.036 [0.027, 0.048] |  |  |
| One child | 11/223 (4.9) | 0.052 [0.028, 0.095] | 1.719 [0.889, 3.324] | .107 | 11/208 (5.3) | 0.056 [0.030, 0.102] | 1.537 [0.786, 3.007] | .209 |
| Two children or more | 14/196 (7.1) | 0.077 [0.045, 0.132] | 2.549 [1.396, 4.654] | .002 | 14/188 (7.4) | 0.080 [0.047, 0.139} | 2.215 [1.198, 4.095] | .011 |
| **Behavioral factors** |  |  |  |  |  |  |  |  |
| **Work situation** |  |  |  | .013 |  |  |  | .040 |
| Not working | 20/564 (3.5) | 0.037 [0.024, 0.057] |  |  | 18/495 (3.6) | 0.038 [0.024, 0.060] |  |  |
| Home office/at work w/o contact | 25/823 (3.0) | 0.031 [0.021, 0.047] | 0.852 [0.469, 1.550] | .600 | 25/784 (3.2) | 0.033 [0.022, 0.049] | 0.874 [0.472, 1.619] | .669 |
| At work with contact | 34/547 (6.2) | 0.066 [0.047, 0.094] | 1.803 [1.024, 3.173] | .041 | 31/516 (6.0) | 0.064 [0.044, 0.092] | 1.694 [0.935, 3.069] | .082 |
| **Adherence to hygiene regulations** |  |  |  |  |  |  |  |  |
| Adherence | 15/604 (2.5) | 0.025 [0.015, 0.043] |  |  | 13/542 (2.4) | 0.025 [0.024, 0.043] |  |  |
| No adherence | 69/1829 (3.8) | 0.039 [0.031, 0.050] | 1.539 [0.874, 2.712] | .135 | 61/1252 (4.9) | 0.051 [0.040, 0.066] | 2.084 [1.135, 3.826] | .018 |
| **Public transportation** |  |  |  |  |  |  |  |  |
| No regular use | 67/1973 (3.4) | 0.025 [0.015, 0.043] |  |  | 58/1380 (4.2) | 0.044 [0.034, 0.057] |  |  |
| Regular use | 17/460 (3.7) | 0.039 [0.031, 0.050] | 1.092 [0.635, 1.877] | .751 | 16/414 (3.9) | 0.040 [0.024, 0.066] | 0.916 [0.521, 1.612] | .762 |

*calculated as estimated marginal means with weighted mean for categorical factors and mean Age (47.39 years)
* the first variable in each category is used as reference unless otherwise specified

**S2 Multicollinearity assessment using tolerance/VIF, Pearson correlation between pairs of continuous variables and between pairs of one continuous and one categorical variable each, and cross-tabulation with chi-square square statistic and effect size as measure of the strength of association between pairs of categorical variables**

| **Coefficients^a^** | | | | |
| --- | --- | --- | --- | --- |
| Model | |  |  |  |
|  |  | Tolerance | VIF |  |
| 1 | (Constant) |  |  |  |
|  | Age | .725 | 1.380 |  |
|  | gender | .992 | 1.008 |  |
|  | Living space per person | .764 | 1.309 |  |
|  | Number of minor children in houshold | .896 | 1.116 |  |
|  | Workplace situation | .840 | 1.191 |  |
|  | Adherence to hygiene regulations | .963 | 1.039 |  |
|  | Public transportation | .957 | 1.045 |  |


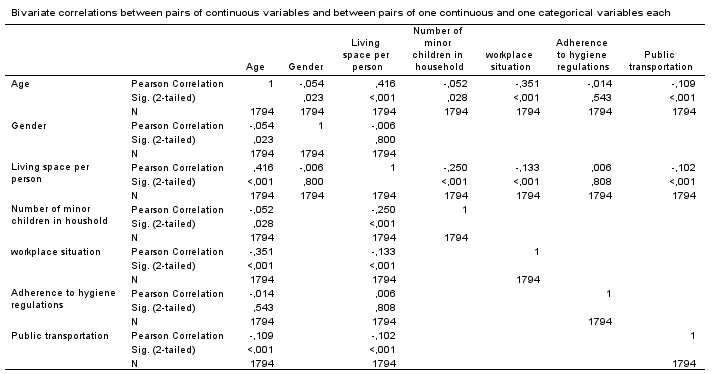


Association between Gender and Number of minor children in household


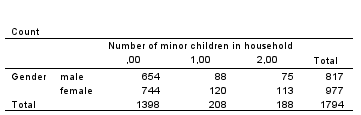


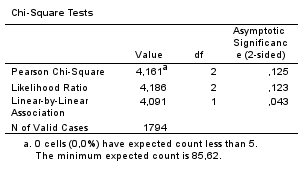


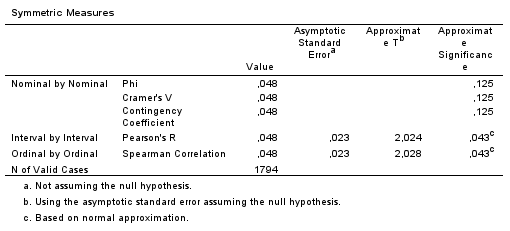


Association between Gender and workplace situation


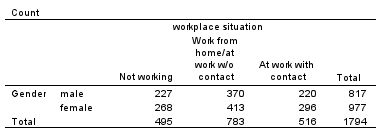


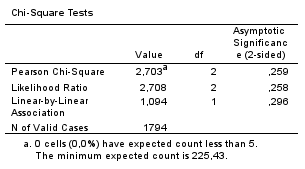


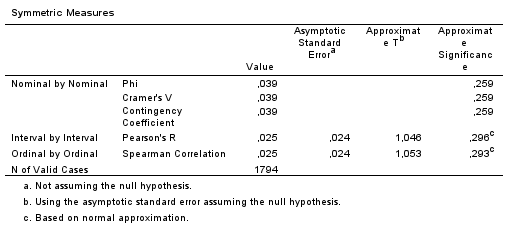


Association between Gender and Adherence to hygiene regulations


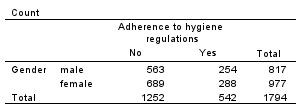


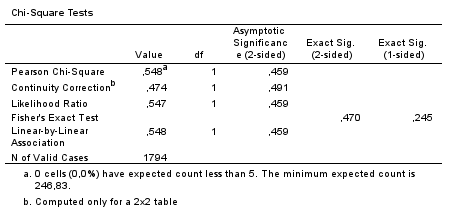


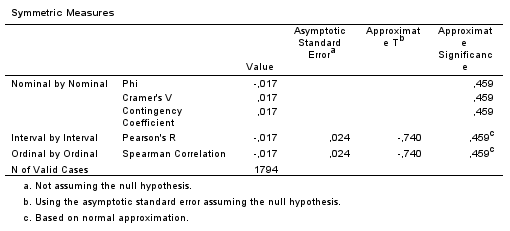


Association between Gender and Regular use of public transportation


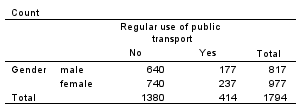


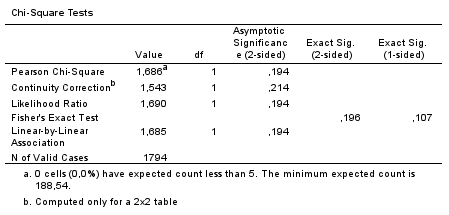


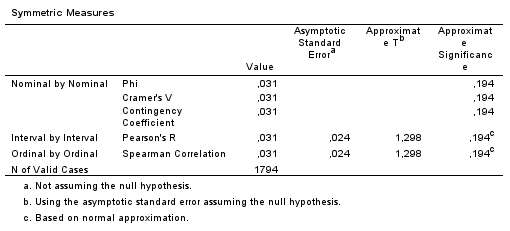


Association between Number of minor children in household and Workplace situation


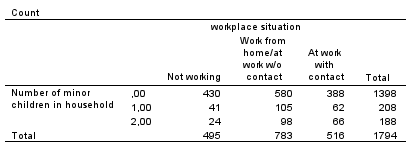


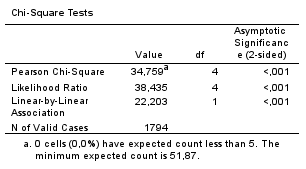


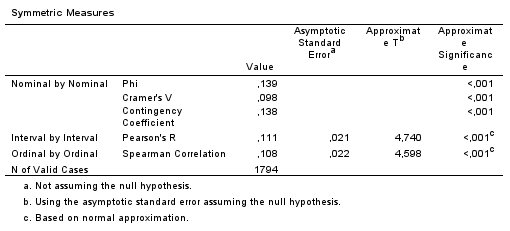


Association between Number of minor children in household and Adherence to hygiene regulations


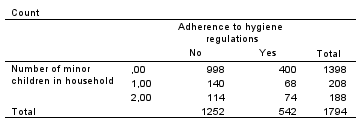


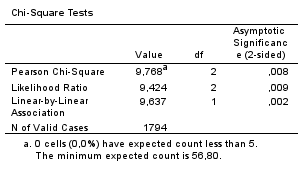


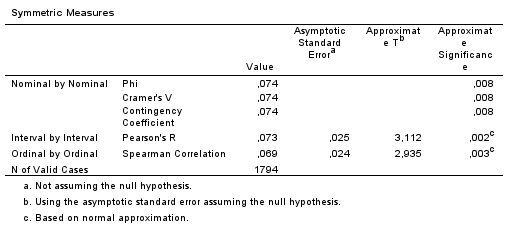


Association between Number of minor children in household and Regular use of public transportation


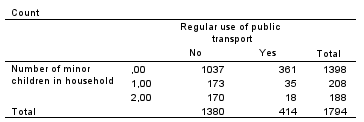


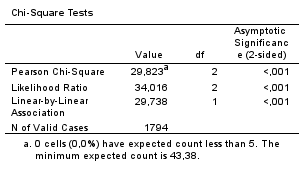


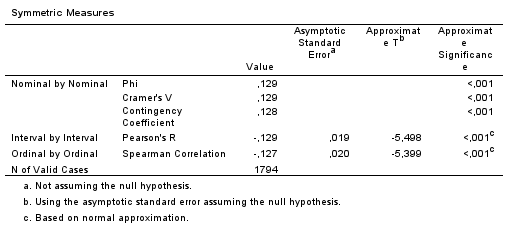


Association between Workplace situation and Adherence to hygiene regulations


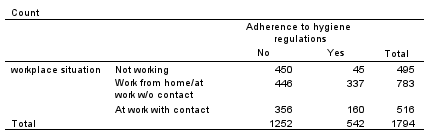


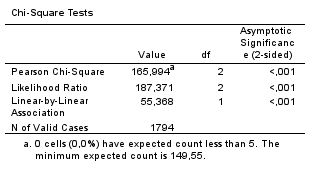


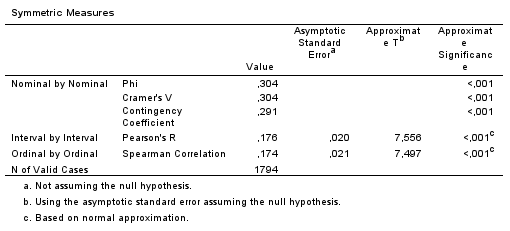


Association between Workplace situation and Regular use of public transportation


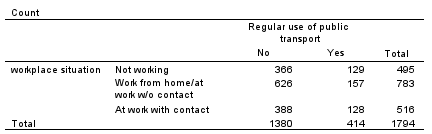


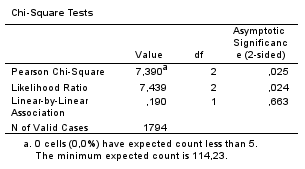


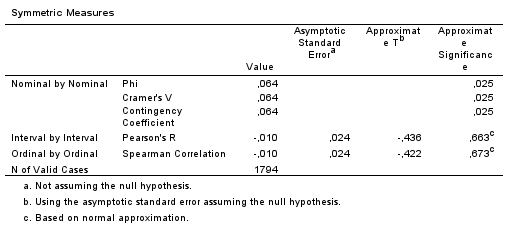


Association between Adherence to hygiene regulations and Regular use of public transportation


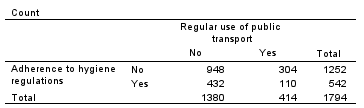


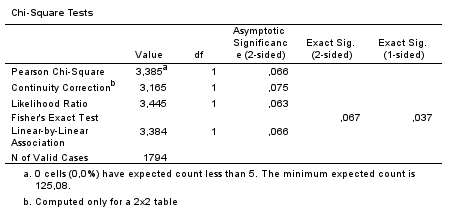


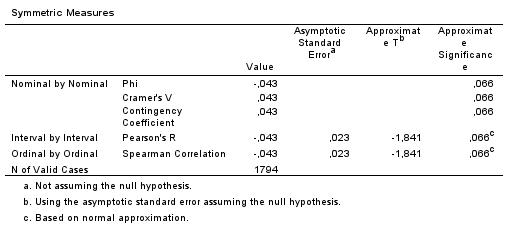


S3 Sociodemographic characteristics and SARS-CoV-2 specific information of the CoCoS study collective (survey completed, n = 2433) and complete cases (N=1794) compared to the potential participants and the general adult Cologne population.

|  | General adult Cologne population | | Potential participants | | CoCoS study sample (survey completed) | | Complete cases | | Risk group*² | |
| --- | --- | --- | --- | --- | --- | --- | --- | --- | --- | --- |
| **Sample** |  |  |  |  |  |  |  |  |  |  |
| Participants (18 yrs. or older) | 913,009 | 100% | 6,000 | 100% | 2,433 | 40.55% | 1,794 | 29.90% | 253 | 4.22% |
| **Gender*** |  |  |  |  |  |  |  |  |  |  |
| Female | 470,577 | 51.54% | 3,086 | 51.44% | 1,312 | 53.93% | 977 | 54.46% | 156 | 61.66% |
| **Age*** |  |  |  |  |  |  |  |  |  |  |
| 18-34 years (%) | 270,538 | 29,63% | 1,662 | 27.70% | 570 | 23.43% | 463 | 25.81% | 40 | 15.81% |
| 35-59 years old (%) | 388,678 | 42,57% | 2,640 | 44.01% | 1125 | 46.24% | 898 | 50.06% | 205 | 81.03% |
| 60-74 years old (%) | 153,890 | 16,86% | 1,095 | 18.25% | 511 | 21.00% | 336 | 18.73% | 8 | 3.16% |
| 75 years or older (%) | 99,903 | 10,94% | 602 | 10.04% | 227 | 9.33% | 97 | 5.41% | 0 | 0% |
| **No. of household members** |  |  |  |  |  |  |  |  |  |  |
| Average | 913,009 | 1.88 | NA | NA | 1,906 | 2.32 | 1,794 | 2.30 | 253 | 3.55 |
| 1-2 | 438,859 | 77.68% | NA | NA | 1,302 | 68.31% | 1,236 | 68.91% | 21 | 8.30% |
| 3-4 | 106,314 | 18.82% | NA | NA | 535 | 28.07% | 492 | 27.42% | 203 | 80.24% |
| 5 or more | 19,800 | 3.50% | NA | NA | 69 | 3.62% | 66 | 3.68% | 29 | 11.46% |
| Missing values^#^ | - | - | - | - | 527 | 21.66% | - | - | 0 | 0% |
| **School education** |  |  |  |  |  |  |  |  |  |  |
| No school leaving certificate | 36,520 | 4.00% | NA | NA | 9 | 0.46% | 8 | 0.45% | 2 | 0.80% |
| Secondary school diploma | 374,333 | 41.00% | NA | NA | 475 | 24.39% | 414 | 23.39% | 36 | 14.34% |
| High school graduation | 502,155 | 55.00% | NA | NA | 1,463 | 75.14% | 1,348 | 76.16% | 213 | 84.86% |
| Missing values^#^ | - | - | - | - | 486 | 19.97% | 24 | 1.34% | 2 | 0.79% |
| **Employment status** |  |  |  |  |  |  |  |  |  |  |
| Student/apprenticeship | 122,849 | 13.46% | NA | NA | 178 | 8.90% | 157 | 8.99% | 0 | 0% |
| Employed | 582,613 | 63.81% | NA | NA | 1,153 | 57.71% | 1,020 | 58.42% | 166 | 65.61% |
| Self-employed | NA | NA | NA | NA | 226 | 11.31% | 185 | 10.60% | 42 | 16.60% |
| Retired | NA | NA | NA | NA | 326 | 16.32% | 285 | 16.32% | 7 | 2.77% |
| Unemployed | 45,225 | 4.60% | NA | NA | 49 | 2.45% | 46 | 2.63% | 4 | 1.58% |
| Other^2^ | NA | NA | NA | NA | 66 | 3.30% | 53 | 3.04% | 34 | 13.44 |
| Missing Values^#^ | - | - | - | - | 435 | 17.88% | 48 | 2.68% | 0 | 0% |
| **Primary citizenship*** |  |  |  |  |  |  |  |  |  |  |
| German | 727,503 | 79.68% | 5,039 | 84.00% | 2,290 | 94.12% | 1,715 | 95.60% | 233 | 92.09% |
| other than German | 185,506 | 20.32% | 960 | 16.00% | 143 | 5.87% | 79 | 4.40% | 20 | 7.91% |
| **Cumulative SARS-CoV-2 cases** |  |  |  |  |  |  |  |  |  |  |
| Start of the pandemic until June 18, 2021 | 46,195 | 5.06% | NA | NA | 84 | 3.45%  [2.73%-4.18%] | 74 | 4.12%  [3.20%-5.05%] | 20 | 7.91%  [4.58%-11.23%] |
| **Vaccination rate until June 18, 2021** |  |  |  |  |  |  |  |  |  |  |
| Vaccinated at least once | 604,692 | 66.23% | NA | NA | 1,591 | 79.95% | 1,369 | 76.92% | 189 | 77.78% |
| Vaccinated twice | 351,674 | 38.52% | NA | NA | 672 | 33.77% | 565 | 32.98% | 69 | 28.40% |
| Not vaccinated | 308,308 | 33.76% | NA | NA | 399 | 20.05% | 344 | 20.08% | 54 | 22.22% |
| Missing Values^#^ | NA | NA | NA | NA | 443 | 18.20% | 81 | 4.52% | 10 | 3.95% |

*Information obtained directly from the population register.

*² Risk group: < 65 years, living with children in the household, non-adherence to hygiene measures

^#^While the percentages on the variables expressed relate to the respondents of the respective questionnaire item, the percentages on the missing values relate to the sample.

^2^ Maternity leave, parental leave, parental leave or other leave of absence, retraining, federal voluntary service, voluntary social/ecological year
